# Modifiable Risk Factors for Alzheimer Disease and Subjective Memory Impairment across Age Groups

**DOI:** 10.1371/journal.pone.0098630

**Published:** 2014-06-04

**Authors:** Stephen T. Chen, Prabha Siddarth, Linda M. Ercoli, David A. Merrill, Fernando Torres-Gil, Gary W. Small

**Affiliations:** 1 Department of Psychiatry and Biobehavioral Sciences and Semel Institute for Neuroscience and Human Behavior, University of California Los Angeles, Los Angeles, California, United States of America; 2 UCLA Longevity Center on Aging, University of California Los Angeles, Los Angeles, California, United States of America; 3 David Geffen School of Medicine, University of California Los Angeles, Los Angeles, California, United States of America; 4 Department of Public Policy, UCLA School of Public Affairs, University of California Los Angeles, Los Angeles, California, United States of America; Nathan Kline Institute and New York University School of Medicine, United States of America

## Abstract

**Introduction:**

Previous research has identified modifiable risk factors for Alzheimer's disease (AD) in older adults. Research is limited on the potential link between these risk factors and subjective memory impairment (SMI), which may precede AD and other dementias. Examination of these potential relationships may help identify those at risk for AD at a stage when interventions may delay or prevent further memory problems. The objective of this study was to determine whether risk factors for AD are associated with SMI among different age groups.

**Method:**

Trained interviewers conducted daily telephone surveys (Gallup-Healthways) of a representative community sample of 18,614 U.S. respondents, including 4,425 younger (age 18 to 39 years), 6,365 middle-aged (40 to 59 years), and 7,824 older (60 to 99 years) adults. The surveyors collected data on demographics, lifestyles, and medical information. Less education, smoking, hypertension, diabetes, less exercise, obesity and depression, and interactions among them, were examined for associations with SMI. Weighted logistic regressions and chi-square tests were used to calculate odds ratios and confidence intervals for SMI with each risk factor and pairwise interactions across age groups.

**Results:**

Depression, less education, less exercise, and hypertension were significantly associated with SMI in all three age groups. Several interactions between risk factors were significant in younger and middle-aged adults and influenced their associations with SMI. Frequency of SMI increased with age and number of risk factors. Odds of having SMI increased significantly with just having one risk factor.

**Conclusions:**

These results indicate that modifiable risk factors for AD are also associated with SMI, suggesting that these relationships occur in a broad range of ages and may be targeted to mitigate further memory problems. Whether modifying these risk factors reduces SMI and the eventual incidence of AD and other dementias later in life remains to be determined.

## Introduction

Alzheimer disease (AD) afflicts an estimated 5.4 million people in the United States—one in eight Americans over age 65—and accounts for approximately $200 billion in direct healthcare costs and $210 billion in unpaid caregiving each year. By 2050, AD prevalence is projected to be 11 million to 16 million [1]. Currently available pharmacologic treatments for AD have demonstrated only modest effects on symptoms and disease progression, and new drug development the last two decades has yielded unsuccessful results [2].

Given the paucity of evidence for effective disease-modifying treatments, and the recognition that the disease process begins years in advance of symptoms, recent attention has focused on the prevention or delay of AD onset through other means, such as changes in lifestyle and treating other health conditions. In 2010, the United States National Institutes of Health systematically reviewed the scientific data on the relationship of multiple factors with cognitive decline and AD [3]. The report identified several potentially modifiable factors associated with altering risk for cognitive decline or AD, or both: diabetes, smoking, depression, cognitive engagement, physical activity, and diet. Barnes and Yaffe reviewed the data on these risk factors, plus obesity and hypertension, to project the effect of risk factor reduction on AD [4]. By calculating population attributable risks, which take into account a given risk factor's prevalence and strength of association with the outcome of interest, they determined that a 10–25% improvement in all seven risk factors—diabetes, hypertension, obesity, smoking, depression, less education, and physical inactivity—could potentially prevent up to 1.1 to 3.0 million cases of AD worldwide and 184,000 to 492,000 in the United States.

Clinical stages that precede AD have been identified. Perhaps the most well studied cohort at risk for developing AD is comprised of older individuals with mild cognitive impairment (MCI), which is characterized by cognitive decline intermediate between normal aging and dementia [5], [6]. Approximately 14–18% of individuals 70 years and older have MCI, 10–15% of whom will progress to dementia each year [7].

Subjective memory impairment (SMI) may be a precursor to MCI. Large-scale longitudinal studies show that individuals with SMI and worry or concern for their memory are at significantly greater risk for MCI and subsequent dementia [8], [9]. While these and other studies have demonstrated that SMI predicts future cognitive decline, including the development of AD and related dementias [10], [11], other studies have not [12]–[14].

Our group previously reported on the relationships between SMI and depressive symptoms [15], a genetic risk factor for AD [16], cerebral glucose metabolism [17], and amyloid and tau brain pathology in non-demented adults [18]. Other groups have found that standardized measures of SMI relate significantly to neuropsychological test performance [19], [20]. We have reported on the positive effects of a six-week educational program including memory training, physical activity, stress reduction, and healthy diet on subjective and objective cognitive measures [21]. Healthy behaviors are associated with better self-perceived memory abilities throughout adult life, suggesting that lifestyle behavior habits may protect brain health and possibly delay the onset of memory symptoms as people age [22].

Subjective memory impairment increases with age [23], [24], with 21% to 26% of older cognitively intact community samples reporting memory problems [25], [26]. The prevalence and importance of SMI in younger populations have not been well studied. In this investigation, we hypothesized that modifiable risk factors for AD have similar relationships with SMI across age groups. If so, earlier risk reduction through lifestyle changes and medical treatment may not only reduce the prevalence and impact of AD, but also of SMI and MCI.

## Methods

### Survey procedures

Gallup Poll Daily tracking interviews of 18,614 US adults, aged 18 years and older, for the period between December 19, 2011, and January 31, 2012, provided the data set for this analysis. Methods and response rates for this Gallup survey were previously described elsewhere [22]. Gallup uses professionally trained interviewers, who have the leeway to terminate calls if the respondent seems unable to understand or respond to the questions. Because this analysis did not pose risk to respondents and used data collected exclusively by the Gallup Organization, the study received a waiver from the UCLA Human Subjects Protection Committee.

### Questionnaire items

The questionnaire included items soliciting information about medical conditions, health-related behaviors, and self-perception of memory (Table 1). The items selected for analysis were based on the variables identified by Barnes and Yaffe as modifiable risk factors for AD. SMI was assessed using a single question about the presence of perceived memory problems. Responses to this question, adapted from a meta-memory questionnaire item, are associated with objective memory performance scores [27].

**Table 1:** Gallup Poll Questions on Study Risk Factors.

Questions	Possible responses
Have you ever been told by a physician or nurse that you have any of the following, or not?	Yes/No
High blood pressure	
Depression	
Diabetes	
In the last seven days, on how many days did you exercise for more than 30 minutes?	0–7 days
Do you smoke?	Yes/No
What is your highest completed level of education?	Less than high school diploma
	High school degree or diploma
	Technical/Vocational school
	Some college
	College graduate
	Post graduate work or degree
What is your approximate weight?	Actual numbers
What is your height in feet and inches?	
Do you have any problems with your memory?	Yes/No

### Statistical analyses

Analyses were performed using SAS 9.3 software, which allows estimation of parameters and hypothesis testing weighted by sample weights to ensure correct computation of standard errors. Respondents were divided into three age groups: younger (18–39 years), middle-aged (40–59 years), and older (60–99 years) adults. The relationships between SMI and risk factors were studied using logistic regression, with presence of SMI as the dependent variable and presence of risk factors as the independent variables. Separate models were computed for each age group. Risk factors examined were less education, smoking, hypertension, diabetes, less exercise, obesity and depression. We defined obesity as a body mass index (BMI)>30), less education as having less than the completion of high school (consistent with Barnes and Yaffe), and less exercise as exercising less than twice a week for 30 minutes or more. The relationships between SMI and risk factors were examined in two ways: 1) using the number of risk factors as the independent variable, with the no risk factor group as the reference group; and 2) using all the individual risk factors and all pairwise interactions between risk factors as independent variables. A single logistic regression was estimated for each age group for each of these analyses.

Because of the large sample size, results can be statistically significant even when actual effect sizes are small. In these analyses, we thus emphasized odds ratios (and the 95% confidence intervals (CI)) over p-values.

## Results

The demographic data and frequencies of potential risk factors and SMI across age groups are presented on Table 2. As noted, the data were weighted to match targets from the U.S. Census Bureau by age, sex, region, education, ethnicity, and race. The older adult group had the highest proportion of women and whites. The frequencies of risk factors varied with age. A greater number of older adults had less education, hypertension, diabetes, and less exercise compared to younger and middle-aged adults. Middle-aged adults had the greatest percentages of obesity and depression. Younger adults had the highest rate of smokers. SMI increased with age: 14.4% of younger adults, 21.9% of middle-aged, and 26.1% of older adults.

**Table 2:** Characteristics of Subjects and Frequencies of Risk Factors and Subjective Memory Impairment according to Age Group.

	All Subjects	Younger	Middle-Aged	Older
	18–99 years	18–39 years	40–59 years	60–99 years
	(n = 18,614)	(n = 4,425)	(n = 6,365)	(n = 7,824)
Age, mean (SD), years	47.1 (18.1)	27.8 (8.0)	49.3 (5.8)	70.1 (6.3)
Education (%)				
Less than high school	11.6	11.8	9.1	14.8
High school graduate	29.2	25.3	29.5	34.3
Some college	28.7	34.1	27.5	23.0
College graduate	17.0	18.2	19.4	12.2
Post graduate	13.0	10.2	14.1	15.1
Women (%)	50.2	47.7	49.3	54.7
Ethnicity (%)				
White	73.1	62.4	75.6	83.9
Hispanic	13.0	20.0	11.1	6.2
African American	10.3	13.2	10.1	6.9
Asian	2.1	3.8	1.4	0.7
Other	1.5	0.6	1.8	2.3
Married (%)	58.9	45.6	69.8	62.3
Monthly income (%)				
$500–2999	39.2	43.3	32.7	42.9
$3000–7,499	40.9	40.1	41.1	41.7
$7,500+	19.9	16.7	26.1	15.5
Risk Factor (%)				
Less education	11.7	11.8	9.1	14.8
Obesity^a^	27.1	21.9	31.5	28.0
Smoking	21.1	24.8	23.8	12.4
Hypertension	28.8	9.2	30.0	53.8
Diabetes	10.7	2.7	10.5	21.9
Depression	17.0	15.0	18.8	17.2
Less exercise^b^	39.0	34.0	41.2	42.9
Subjective Memory Impairment (%)	20.3	14.4	21.9	26.1

a Obesity defined as Body Mass Index >30.

b Exercise 30 minutes or more < twice a week.

**Figure 1:**
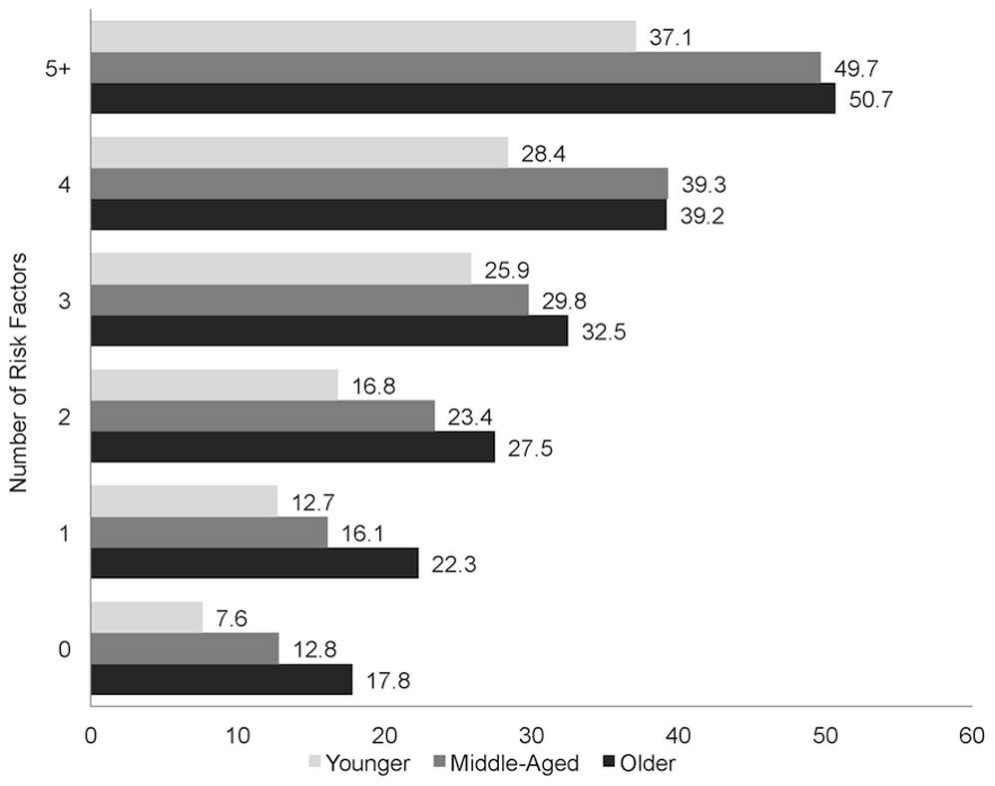
Frequencies (%) of Subjective Memory Impairment according to Number of Risk Factors across Age Groups.

**Figure 2:**
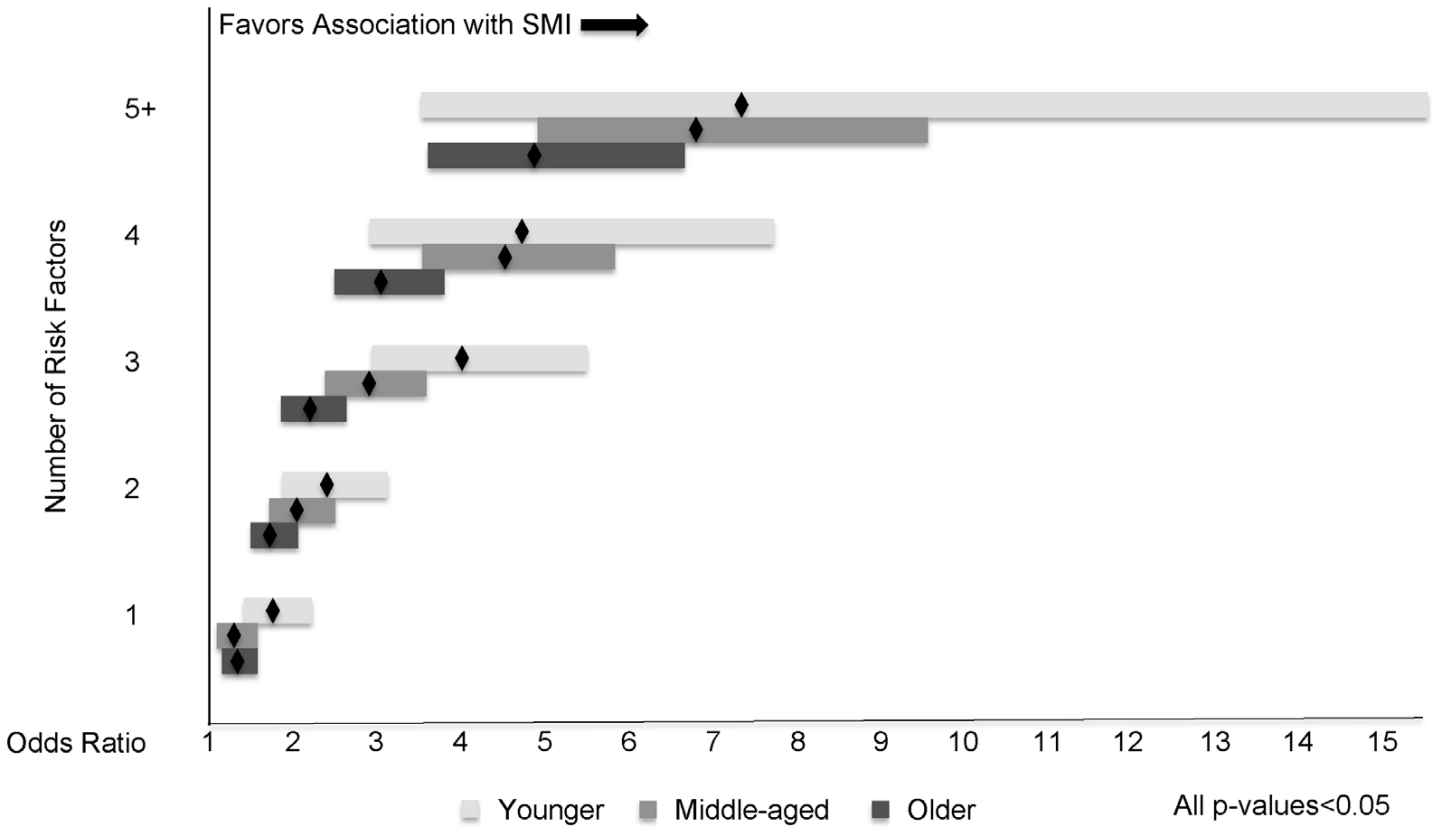
Odds Ratios and 95% Confidence Intervals for Subjective Memory Impairment (SMI), Number of Risk Factors vs. No Risk Factors, across Age Groups.

Across all age groups, SMI generally increased with the number of risk factors (Figure 1). The odds of having SMI were significantly greater with having just one risk factor than having none, and increased with the number of risk factors (Figure 2). Though the younger and middle-aged groups reported SMI at lower frequencies than the older group, for a given number of risk factors, the odds ratios for SMI were generally highest in the younger group, followed by the middle-aged group.

**Figure 3:**
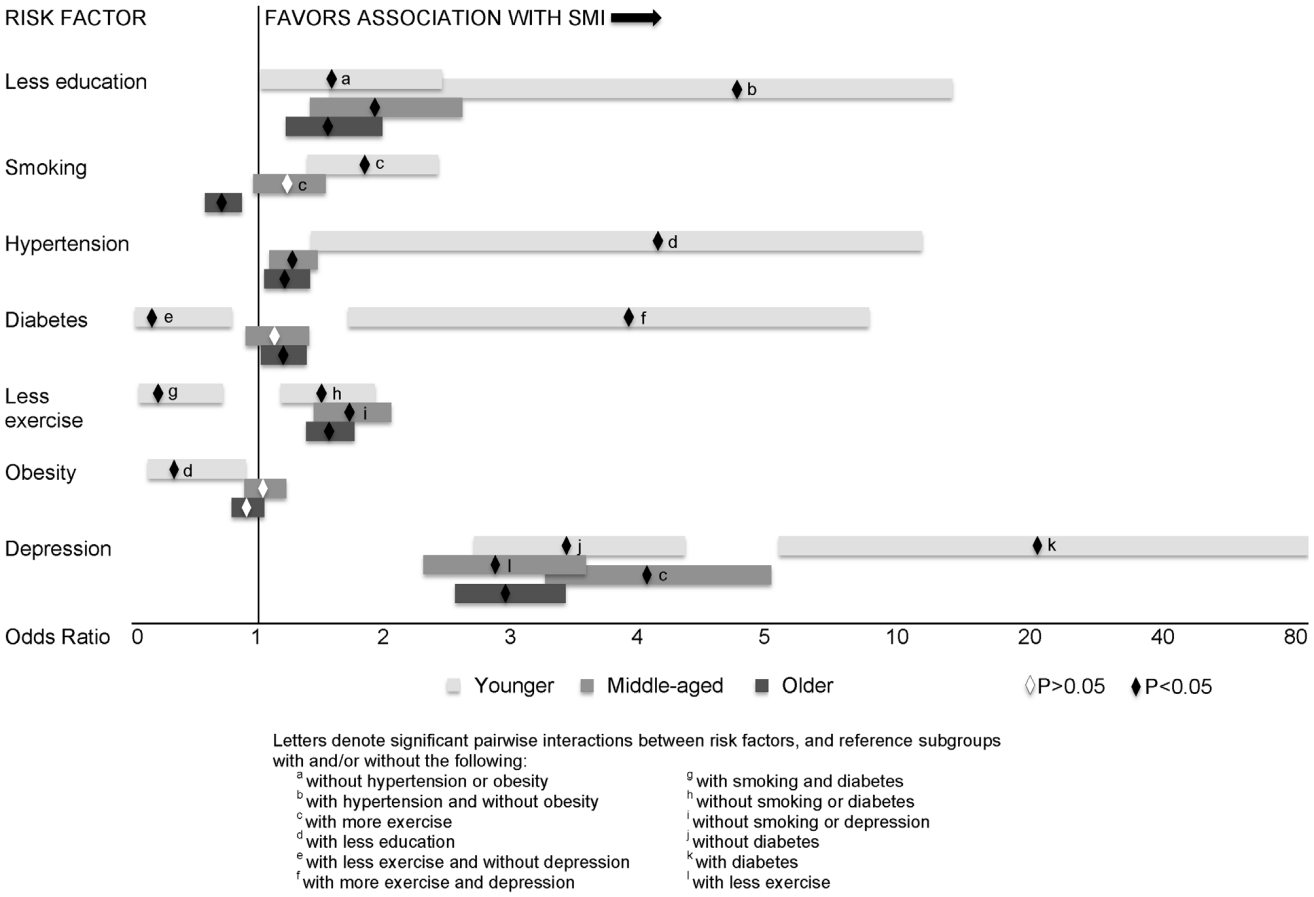
Odds Ratios and 95% Confidence Intervals for Subjective Memory Impairment (SMI) with each Risk Factor across Age Groups.

Odds ratios for having SMI with each potential risk factor are shown in Figure 3. Less education, less exercise, hypertension, and depression significantly increased the odds for having SMI in all age groups. Depression was associated with the greatest odds ratios for SMI in all age groups. There were several significant interactions between risk factors among younger and middle-aged adults, but none among older adults. Only significant interactions are shown in Figure 3.

Among younger adults, significant interactions were found between diabetes and depression (χ^2^(1) = 6.74, p = 0.04), education and obesity (χ^2^(1) = 4.05, p = 0.04), education and hypertension (χ^2^(1) = 3.75, p = 0.05), smoking and exercise (χ^2^(1) = 6.51, p = 0.01), and diabetes and exercise (χ^2^(1) = 4.53, p = 0.03). Some interactions had additive or synergistic effects on SMI, while others had opposing or contradictory effects. Among younger adults with diabetes, those with depression as well were 21.8 times (95% CI: [5.5, 86.0]) more likely to report SMI than those without depression. For respondents without diabetes, those with depression as well were 3.4 times (95%CI: [2.7, 4.4]) more likely to report SMI than those without depression. Among less educated younger adults, those with hypertension were 4.1 times (95%CI: [1.4, 12.2]) more likely to report SMI than those without hypertension, while those with obesity were *less* likely to report SMI than non-obese subjects (OR = 0.3, 95%CI: [0.1, 0.9]). These odds ratios were not significant in younger subjects with more education. Among respondents who exercised more, smokers were 1.8 times more likely (95%CI: [1.4, 2.4]) to have SMI than non-smokers. Interestingly, among those who exercised less, smoking did not have a significant association with SMI. Diabetes was associated with greater SMI among younger adults who had depression but exercised more (OR = 3.9, 95%CI: [1.7, 9.0]), but with less SMI among those with no depression and less exercise (OR = 0.16, 95%CI: [0.03, 0.8]). Less exercise was associated with greater SMI among younger nonsmokers without diabetes, but with less SMI among younger smokers with diabetes.

In middle-aged adults, significant interactions were found between smoking and exercise (χ^2^(1) = 4.07, p = 0.04), and exercise and depression (χ^2^(1) = 4.89, p = 0.03). Among nonsmokers without depression, those who exercised less were 1.7 times (95% CI: [1.4, 2.0]) more likely to report SMI than those who exercised more. Depression was associated with greater SMI regardless of frequency of exercise, but more strongly related in those who exercised more (OR = 4.1, 95%CI: [3.3, 5.0]) than in those who exercised less (OR = 2.9, 95%CI: [2.3, 3.6]).

## Discussion

This study demonstrates that some of the established modifiable risk factors for AD—less education, less exercise, hypertension, and depression—also increase the odds for having SMI, which may be a precursor to AD and other dementias. While studies of risk factors for AD generally involve older individuals, this study found some of the same modifiable risk factors to increase the likelihood of SMI consistently across age groups. These findings may be expected, or at least suspected, in the older adult group. However, that these same relationships were found in the younger and middle-aged groups raises interesting questions. Do these risk factors earlier in life increase risk for not only SMI, but for MCI and dementia? Does mitigating these risk factors also mitigate future risk for SMI and MCI, as well as dementia? Does SMI, in certain cases, represent the earliest clinical marker for AD or dementia?

Our results indicate that younger and middle-aged adults report SMI at relatively high frequencies. Possible explanations are that people may have a lower tolerance for memory difficulties when they are younger [28], and younger people have more tasks and responsibilities that require a higher level of memory performance [29]. Other studies have shown that young adults tend to ascribe their memory complaints to temporary extrinsic factors, such as emotional problems and stressful life events, whereas older people more often mentioned persistent and intrinsic reasons such as aging [23], [24]. Despite these differences in self-ascription, our finding that different age groups share similar risk factors for SMI suggests that common mechanisms may promote SMI at any age. Subjective memory impairment in younger adults is unlikely to be related to AD, though neurobiologic changes in AD can occur decades before the onset of clinical symptoms, and may play a plausible role in mediating SMI later in life. Longitudinal studies that include early biomarkers for AD are needed to elucidate the significance of SMI at younger ages over time.

This study identified several pairwise interactions between risk factors that significantly affected their relationships with SMI in the younger and middle-aged groups. These results suggest that the associations between risk factors and SMI in these age groups were significantly influenced by other risk factors. The number of interactions between risk factors in the younger adult group and their varied effects on SMI make it difficult to make any particular conclusions regarding relationships between individual risk factors and SMI in this age group. In the older adult group, none of the associations was affected by interactions between risk factors, suggesting that the risk factors are associated with SMI in this age group independent of the effects of other risk factors.

Across all age groups, the number of risk factors, regardless of the type of risk factor, increased the odds for SMI. Having just one risk factor, again without regard for the type of risk factor, increased the odds for SMI over having no risk factor. These data suggest that having any of these risk factors, particularly in combination, significantly increases one's risk for SMI.

Among the risk factors studied, depression was the most strongly associated with SMI across all age groups. This finding is consistent with a vast literature that links depression with memory disorders, though the relationships are quite complex and intricate. Strong evidence supports depression as both a risk factor for dementia [30], [31] as well as a prodromal phase of dementia [32]. Either may be the case, depending on the type of depression. Early-onset and recurrent depression may constitute long-term risk factors for development of dementia, whereas the onset of depressive symptoms later in life may reflect a prodromal phase of dementia [33]. A greater number of depressive episodes may increase the risk for dementia [34], while continued long-term treatment with certain antidepressants may reduce the rate of dementia [35]. The association between depression and SMI in this study must be interpreted with caution. Self-reported diagnosis of depression may not be as reliable or accurate as professionally reported diagnoses, or may include other conditions not clinically recognized as depression. Nonetheless, the strong association merits further investigation, as potential implications may impact risk reduction for dementia.

Less education was associated with SMI in all age groups in this study, though interactions with hypertension and obesity limited this association in younger adults. Previous studies have shown that lower levels of education are associated with high blood pressure [36] and obesity [37]. Education is a key element of “cognitive reserve,” or the ability to compensate for pathology through more efficient utilization of, or enhanced ability to recruit, alternate brain networks developed by complex mental activity [38]. Worldwide, less education potentially contributes to the greatest proportion of AD cases.[4] Fortunately, some randomized clinical trials (RCTs) have shown that cognitive training interventions in healthy older people improve specific cognitive domains and daily functioning [39], [40], though none has yet to demonstrate a treatment that delays or prevents dementia in this population [41].

Hypertension was associated with SMI in all age groups. Hypertension in midlife, but not in late life, is associated with increased risk for AD and dementia [42], [43]. Studies do not show any consistent effect of hypertension treatment on the incidence of dementia [44]–[46], though one meta-analysis showed significantly less cognitive decline among the treatment group than placebo group [44]. Whether early or midlife hypertension is a risk factor for late life SMI, and its treatment mitigates such risk, remains to be determined.

Less exercise was associated with increased odds for SMI in all age groups. Exercise interacted with smoking and diabetes in younger adults, and with smoking and depression in middle-aged adults. Unhealthy lifestyle habits such as physical inactivity and smoking are related to each other as well as to diabetes and depression [47], [48]. A recent meta-analysis of sixteen prospective studies determined that older individuals who were physically inactive were at significantly increased risk for AD and all-cause dementia [49]. Another review of 24 prospective studies that included a wider range of cognitive outcomes found that physical inactivity was associated with an increased risk of cognitive impairment in all but four of the studies [50]. RCTs have reported that aerobic exercise interventions result in improved cognitive function, particularly motor functioning and auditory attention, in cognitively unimpaired elderly participants [51]. To our knowledge, there is no published RCT to determine whether an exercise intervention can delay or prevent AD, though one RCT did report that older individuals with SMI showed modest improvement in cognition after a six-month exercise program [52].

Smoking, another cardiovascular risk factor, did not increase odds for SMI except in a subgroup of younger adults. Smoking actually was associated with lower rates of SMI in older adults. The data on smoking as a risk factor for dementia are mixed. Two meta-analyses concluded that case control studies showed lower or no increased risk for AD and dementia with smoking, whereas prospective cohort studies showed smoking to be a significant risk factor for dementia [53], [54]. A meta-analysis of 19 prospective cohort studies of older adults showed that current smokers have increased risks of dementia and cognitive decline over former smokers and lifetime nonsmokers [55]. Our finding that older current smokers do not report SMI at higher rates than current nonsmokers is consistent with the case control studies, but not with the prospective cohort studies, suggesting that the outcomes are influenced by methodology.

Despite being risk factors for cardiovascular disease and AD, obesity and diabetes were not robustly associated with SMI in this study. Like hypertension, obesity in midlife, but not in late life, is consistently associated with increased risk for AD and dementia [42], [43], [56]. Later in life, a higher BMI may be protective against the development of dementia, while a decrease in BMI may be a marker of incipient dementia [57], [58]. We found no treatment studies that examined purposeful weight loss and incidence of dementia. Whether early or midlife obesity is a risk factor for late life SMI, and its treatment mitigates such risk, remains to be determined. A meta-analysis of nine prospective studies determined that obesity and diabetes significantly and independently increase risk for AD [59]. A 2002 Cochrane review searched for but found no RCTs that examined the cognitive effects of treating type II diabetes [60]. A subsequent review of RCTs evaluating drug treatment effects for cardiovascular risk factors on the incidence of dementia or cognitive decline yielded only studies in which incident dementia or cognitive decline were secondary outcome measures, and little evidence for a preventive treatment effect aimed at vascular risk factors on cognitive decline and dementia [61].

The primary limitation of this study is its cross-sectional, survey-based design. All responses were based on subjects' report, including the primary dependent variable, SMI, which may be confounded by psychological distress and anxiety [62], and thus reflect subjects' feelings at a point in time rather than a persistent state. Previous studies, however, have demonstrated that SMI is associated with measures that are less likely to be transient, including objective cognitive impairment [19], [20] and decline [10], [11], and AD biomarkers in older non-demented persons [16]–[18]. Thus, SMI could reflect preclinical AD brain pathology and suggest the presence of a prodromal stage of AD in older individuals, though not likely in younger persons. The study used non-clinician surveyors, who were trained to identify respondents having difficulty comprehending or responding to questions, but not those with subtle cognitive impairment. Inclusion of subjects with objective cognitive impairment, though known to be associated with SMI, may have influenced our results, particularly as the ages of the respondents increase. The survey's simple, single questions may have limited the clinical validity of the responses. Because of the study's cross-sectional design, we cannot comment on whether having AD risk factors earlier in life increases risk for subsequent SMI, as it does for AD. Rather, our results indicate that these AD risk factors are associated with a higher likelihood of having SMI at one point in time across different age groups. Moreover, the cross-sectional design of the study prohibits any inference of causality, i.e., whether these associations reflect the risk factors causing SMI or vice versa cannot be determined.

Strengths of the present study are that the sample is representative of the U.S. population in terms of age, gender, level of education and income, and ethnicity, and is large enough to provide meaningful results in different age groups. The study suggests common influences of modifiable health and lifestyle factors on subjective memory as on AD. Using this same study sample, we have previously shown that healthy behaviors such as exercise and healthy eating are also associated with less SMI [22]. We cannot determine from this study whether or not these similarities reflect common etiologic mechanisms between AD and SMI. However, if they do reflect common mechanisms, future investigations may exploit these findings to identify individuals at risk for memory symptoms and disorders at a stage early enough to prevent or delay the onset of dementia and to develop effective interventions and public policy. While our studies do not directly address broader policy and program concerns, our results and the future use of the Gallup Healthways data may provide important support for the nation's efforts to improve health care policy by promoting and reinforcing healthy lifestyle behaviors. The 2010 Affordable Care Act (ACA) already attempts a paradigm shift to healthy behaviors and proactive wellness measures to achieve better individual and societal health and reduce medical expenditures. Our study provides further evidence that promoting cognitive/educational training and cardiovascular health and its potential impact on memory falls within the mission of the ACA and other comprehensive health care programs.

This study further establishes relationships between SMI and modifiable risk factors for AD and other dementias. However, we cannot conclude or even infer from the data that these relationships necessarily link SMI to AD. Three of the risk factors for AD—smoking, diabetes, and obesity—were not or weakly associated with SMI in the current study. Several of these relationships were affected by interactions between risk factors, some of which were contradictory, thus weakening the application of the Barnes and Yaffe model for AD risk factors to SMI. The evidence for preventing or delaying AD through identification and modification of risk factors is lacking. Our review of the literature reveals a tremendous need for studies that examine the effects of modifying these risk factors on the incidence of AD and dementia. While there are genetic and other biologically immutable risk factors for dementia, this study shows that there are several potential risk factors that individuals and the public can modify to possibly reduce their risk for SMI. Whether mitigating SMI through these risk factors might lead to reducing the burden of AD and dementia remains a question that, if answered, could influence many lives and health care policy.
